# Validity and reliability of a "simplified" version of the Taylor Complex Figure Test for the assessment of older adults with low formal education

**DOI:** 10.1590/S1980-57642016DN10100010

**Published:** 2016

**Authors:** Jonas Jardim de Paula, Mônica Vieira Costa, Giovanna de Freitas de Andrade, Rafaela Teixeira Ávila, Leandro Fernandes Malloy-Diniz

**Affiliations:** 1 INCT de Medicina Molecular, Faculdade de Medicina, Universidade Federal de Minas Gerais, Belo Horizonte MG, Brazil; 2 Departamento de Psicologia, Faculdade de Ciências Médicas de Minas Gerais, Belo Horizonte MG, Brazil; 3 Laboratório de Investigações em Neurociência Clínica, Universidade Federal de Minas Gerais, Belo Horizonte MG, Brazil; 4 Departamento de Saúde Mental, Faculdade de Medicina, Universidade Federal de Minas Gerais, Belo Horizonte MG, Brazil

**Keywords:** Alzheimer's disease, complex figure test, low formal education, neuropsychological assessment, mild cognitive impairment, doença de Alzheimer, teste figura complexa, educação formal baixo, avaliação neuropsicológica, comprometimento cognitivo leve

## Abstract

**Objective:**

The assessment of visuospatial abilities and memory using tasks such as the Taylor Complex Figure Task (TCFT) is biased for older adults with low formal education. We devised a "Simplified" version of the TCFT designed to assess older adults with low educational background and provide preliminary evidence of its psychometric properties.

**Methods:**

We evaluated a heterogeneous sample of healthy older adults and patients with mild cognitive impairment and Alzheimer's disease dementia using the simplified TCFT and other neuropsychological measures.

**Results:**

Our results suggest that the test copy, immediate and delayed recall have high inter-rater agreement and internal consistency, significant correlations with other tests of visuospatial abilities, memory and intelligence, and also detected significant group differences between controls and patients.

**Conclusion:**

Our study presents a new measure for assessing low-educated elderly with promising evidence of validity and reliability.

## RESUMO

**Objetivo:**

A avaliação de habilidades visioespaciais e de memória por tarefas como a Figura Complexa Taylor (FCT) é enviesada para de idosos com baixa escolarização formal. Propomos uma versão "simplificada" da FCT (FCTs) projetada para a avaliação dos idosos com baixo nível educacional e apresentamos evidências preliminares de suas propriedades psicométricas.

**Métodos:**

Avaliamos uma amostra heterogênea de idosos saudáveis e pacientes com comprometimento cognitivo leve e demência por Doença de Alzheimer usando a FCTs e outras medidas neuropsicológicas.

**Resultados:**

Nossos resultados sugerem que a cópia de teste, a evocação imediata e a evocação tardia têm alta concordância entre avaliadores e elevada consistência interna, apresentam correlações significativas com outros testes de habilidades visioespaciais, memória e inteligência, além de documentarem diferenças significativas entre o grupo controles e os pacientes.

**Conclusão:**

Nosso estudo apresenta uma nova medida para a avaliação neuropsicológica de idosos com baixa escolaridade, com evidências promissoras de validade e confiabilidade

## INTRODUCTION

Several neuropsychological tests have been developed and validated for assessing global cognition, memory, language and executive functions in older adults with low formal education. However, there is a lack of tests related to visuospatial abilities for the clinical assessment of these patients. Visuospatial abilities is a heterogeneous cognitive domain related to the perception, processing and manipulation of spatial content^1^ Visuospatial abilities disturbances are important markers for several clinical conditions, including Dementia, both cortical (Alzheimer's disease dementia - AD, Lewy Bodies Dementia) and subcortical (Parkinson's disease dementia, subcortical vascular dementia) types.^2^

The most common method of assessing visuospatial abilities is the use of drawing tasks. These tests, such as the CERAD Neuropsychological Battery^3^ and the Mattis Dementia Rating Scale, are a relevant part of several structured batteries used for the diagnosis of dementia.^4^ Complex Figure Tests (CFT), the most well-known of which is the Rey-Osterrieth Complex Figure Test,^5^^,^ 6 are often used in clinical practice for the assessment of visuoconstructional abilities and memory recall. Although their role in neuropsychological assessment has been well established in populations with relatively high formal education (8 years or more), results for these tests in adults with low formal education are conflicting. A seminal study on the role of formal education in cognitive performance^7^ reported a large discrepancy on the copy component between subjects with around 12 years of formal education (who typically score above 30 points, out of a maximum score of 36) and illiterates (who usually score between 15 and 20 points on the Rey-Osterrieth Complex Figure Test). Drawing tasks may therefore lose their power to discriminate between clinical and non-clinical samples when educational level is low.^8^ Even when applying specific interpretation parameters stratified by formal education, the use of complex drawing tasks requires special care. In general, participants with low formal education exhibit negative emotional reactions while performing complex figure tasks, affecting motivation.^9^

We believe that "simplifying" a complex drawing task, while maintaining its validity and reliability, is desirable for the assessment of older adults with low formal education. The simpler test might be less susceptible to formal education biases and prove more accessible to participants with low formal education. Thus, the aim of the present study was to adapt and assess the psychometric characteristics (reliability and construct validity) of a "Simplified" version of the Taylor Complex Figure Test (sTCFT). 

## METHODS

**Participants.** We assessed a sample of 189 patients in a unit specialized in the assessment of older adults in the city of Belo Horizonte, Brazil. In the overall sample, 26 older adults had no evidence of cognitive and functional deficits and were labelled "Normal Aging" (NA) participants. We also investigated the performance of three clinical groups: single domain amnestic mild cognitive impairment (aMCI) (N = 42); multiple-domain amnestic mild cognitive impairment (mdaMCI) (N = 48), diagnosed by the Winblad et al. criteria;^10^ and a group of mild AD patients (N = 73), diagnosed according to criteria of McKhann et al.^11^

The participants underwent neuropsychological assessment including the Mini-Mental State Examination, the Mattis Dementia Rating Scale, subtests from the CERAD neuropsychological battery and other neuropsychological tests validated for older adults with low formal education.^12^ We defined the diagnosis by consensus, including the opinion of different professionals. The present study was part of a larger project, which aims to assess the relationship between depression and dementia, approved by the Universidade Federal de Minas Gerais Ethics Board (registry 04/334). The participants or their caregivers, in case of demented patients, gave written consent for participation in the study. 

**Figure 1.**
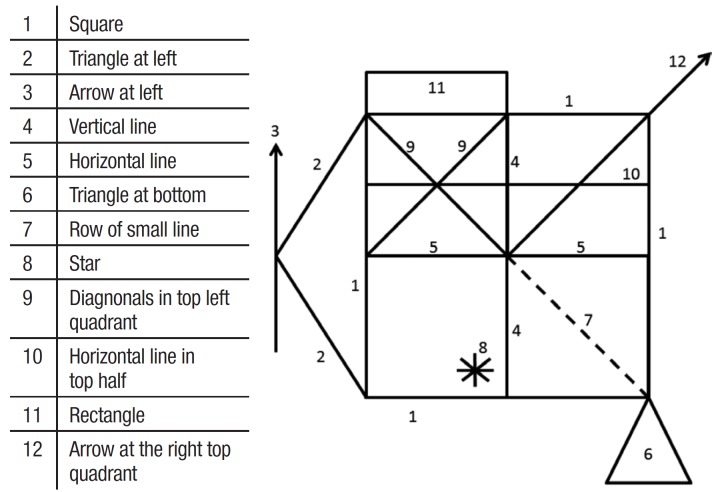
The "Simplified" Taylor Complex Figure Test.

**"Simplified" Taylor Complex Figure Test.** The original Taylor Complex Figure test consists of 18 graphic-elements related to more general-structural components and other more specific-detailed ones. Readers should refer to the original reference for the test stimuli.^13^ For the development of the simplified version, two neuropsychologists analyzed the original figure and sought to reduce its components. The element exclusion criteria involve curves and superimposed elements, two characteristics involved in the "loss of perspective" errors, usually exhibited by patients with low formal education on drawing tests.^14^ We excluded the following elements, represented by their numbers on the original stimuli: Circle,^9^ Semicircle,^12^ Triangle Line,^13^ Horizontal Line between Dots,^15^ and Curves & Cross Lines.^17^ We also modified item 14 (Row of Dots) to a Row of Small Lines. The remaining 12 items are depicted in Figure 1. The stimuli are available from the authors upon request. 

The application procedure involves a non-timed copy trial, followed by a 3-minute immediate recall and a 25-30 minute delayed recall. The scoring procedures were based on the Osterrieth procedure for the Rey Complex Figure Test^6^ for each element: correct (precision) and placed properly (2 points), correct (precision) and placed poorly (1 point), distorted/incomplete placed poorly (0.5 points), absent or not recognizable (0 points). The score ranges from 0 to 24 points. 

**Neuropsychological assessment.** The participants underwent comprehensive neuropsychological assessment by certified clinical neuropsychologists or psychology undergraduates under direct supervision of two of the authors (JJP and LFMD). We selected the assessment protocol based on previous research findings regarding the applicability and validity of different neuropsychological tests for the assessment of Brazilian older adults with low formal education.

Intelligence: To avoid assessment biases due to low formal education of participants we selected a non-verbal fluid intelligence test. The Colored Raven Progressive Matrices.^15^ This test was used as an estimate of participant intelligence. The test has been validated for use in the Brazilian population.^16^

Visuospatial abilities: The Stick Design Test^17^ was adopted, a visuoconstructional test validated for older adults with low formal education.^18^ We also selected the DRS "Constructional Praxis" subscale,^4^ which involves simple drawings, as another measure of this cognitive domain. 

Episodic memory: This domain was assessed by the Brazilian-Portuguese version of the Rey Auditory-Verbal Learning Test,^19^ previously validated for the assessment of low educated older adults.^20^ We used the immediate and delayed recalls (RAVLR IR and DR) as measures of episodic memory, as well as the DRS "Memory" subscale.

**Statistical procedures.** Age, education and depressive symptoms of the four groups were assessed using univariate analysis of variance (Sidak Post hoc test for multiple comparisons) while sex was evaluated by the Chi-square test. The participants differed for age (F(3,188) = 3.47, p = 0.017) and formal education (F(3,188) = 2.86, p = 0.038) but not for sex (c² = 1.89, p = 0.594). The post hoc analysis suggested differences between controls and AD on these variables, albeit with low effect sizes. Nonetheless, due to these differences we compared the performance on neuropsychological tests by a multivariate general linear model containing the cognitive measures as dependent variables, group as a factor, and both age and education as covariates. The Sidak procedure was adopted to compare the estimated marginal means of the sTCFT among the four groups. 

Inter-rater reliability of the copy, immediate and delayed recall components on each test item and the total scores was assessed by calculating the Kappa coefficient of a randomly selected subsample (n = 20) of participants. The test internal consistency was estimated by the Cronbach's alpha. Partial correlations of the sTCFT with other measures of visuospatial abilities and episodic memory were computed, controlling for the effects of age, education and depressive symptoms in order to assess convergent and divergent validity. The coefficient of determination (r²) showed the shared variance between tests. 

## RESULTS

**Figure 2.**
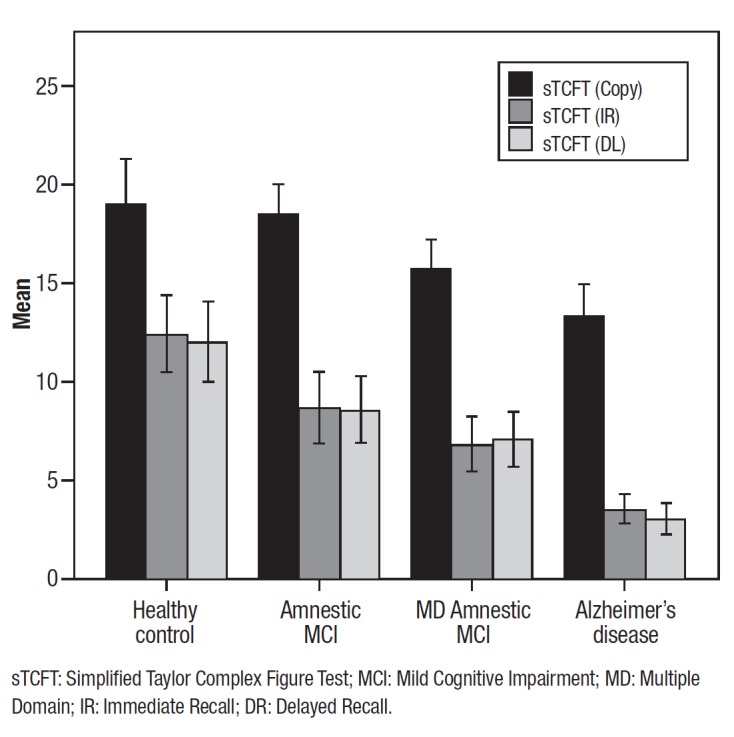
Performance of normal aging older adults and clinical groups on the "Simplified" Taylor Complex Figure Test.

Participants' clinical characteristics are shown in Table 1. Group comparisons suggested a progressive pattern of impairment, with AD patients having lower scores than MDaMCI with patients in this latter group performing worse than both aMCI patients and control subjects. On the sTCFT, significant differences were found for Copy, where the NA and aMCI groups performed better than the MDaMCI and AD groups (all p < 0.05). On the immediate and delayed recalls, the NA outperformed the clinical groups; aMCI and MDaMCI performed better than AD but did not differ to each other. Figure 2 depicts the group performances.

**Table 1.** Participant characteristics and group comparisons on sociodemographic and cognitive measures.

**Measures**	**NA (n = 26)**		**aMCI (n = 42)**		**MD-aMCI (n = 48)**		**AD (n = 73)**		**Group differences¹**	
	**Mean**	**SD**	**Mean**	**SD**	**Mean**	**SD**	**Mean**	**SD**	**F**	**n²**
Age	71.92	7.83	74.57	7.58	77.10	8.59	77.08	7.71	-	-
Education	5.69	4.66	5.36	4.96	3.65	3.25	3.81	3.44	-	-
DRS - Total Score	129.10	10.60	122.11	9.31	112.54	8.08	98.38	13.34	71.47**	0.54
Raven Progressive Matrices	21.23	6.31	20.71	6.40	16.85	4.36	15.45	4.78	9.73**	0.14
Stick Design Test	11.85	0.46	11.69	0.81	10.98	1.41	9.68	2.41	14.69**	0.19
DRS - Constructional Praxis	5.42	0.99	5.21	1.52	4.68	1.52	4.12	1.90	4.21**	0.06
RAVLT IM	7.96	2.24	3.69	2.21	3.37	2.16	2.11	1.80	49.78**	0.45
RAVLT DR	7.88	2.23	3.86	2.25	3.46	2.32	1.86	1.85	49.95**	0.45
DRS - Memory	22.99	2.04	18.59	3.00	17.03	2.91	13.15	3.47	67.91**	0.53
sTCFT Copy	19.12	5.38	18.50	5.08	15.79	5.09	13.34	6.64	6.19**	0.10
sTCFT Immediate Recall	12.46	4.62	8.71	5.82	6.91	4.80	3.61	3.25	24.91**	0.30
sTCFT Delayed Recall	12.08	4.90	8.62	5.62	7.17	4.57	3.11	3.32	28.81**	0.33

^1^Multivariate General Linear Model with age and education as covariates. *< 0.01; **< 0.001. DRS: Dementia Rating Scale; RAVLT: Rey Auditory-Verbal Learning Test; IM: Immediate Recall; DR: Delayed Recall; sTCFT: "Simplified" Taylor Complex Figure Test; NA: Normal Aging; aMCI: Amnestic Mild Cognitive Impairment; MDaMCI: Multiple Domain Amnestic Mild Cognitive Impairment; AD: Alzheimer's disease.

sTCFT: Simplified Taylor Complex Figure Test; MCI: Mild Cognitive Impairment; MD: Multiple Domain; IR: Immediate Recall; DR: Delayed Recall.

Table 2 shows the results of the inter-rater reliability analysis. When each individual element of the Copy and Recall trials were judged by two different examiners (G.F.A. and M.V.C), there was substantial agreement for most of the test items (Kappa > 0.6). Only one element on the Copy condition had a fair agreement between the judges (Row of Small Lines, Kappa = 0.36). When the total scores were analyzed, the non-parametric correlations of judges' scores were all significant, with high effect sizes: Copy (r = 0.89, p < 0.001, R² = 79%), Immediate Recall (r = 0.97, p < 0.001, R² = 94%), and Delayed Recall (r = 0.96, p < 0.001, R² = 92%). The Copy (0.912), Immediate Recall (0.846) and Delayed Recall (0.857) test components showed a high internal consistency.

**Table 2.** Inter-rater agreement (Kappa index) of "Simplified" Taylor Complex Figure Test scoring.

**Element**	**Copy**	**Immediate Recall**	**Delayed Recall**
1 - Square	0.84	0.78	0.77
2 - Triangle at left	0.76	0.74	0.77
3 - Arrow at left	0.82	0.91	0.83
4 - Vertical line	0.77	0.74	0.71
5 - Horizontal line	0.64	0.63	0.73
6 -Triangle at bottom	0.53	0.93	0.92
7 - Row of small lines	0.36	0.73	0.64
8 - Star	0.65	0.67	0.67
9 - Diagonals in top left quadrant	0.66	0.76	0.76
10 - Horizontal line in top half	0.54	0.58	0.79
11 - Rectangle	0.75	0.79	0.89
12 - Arrow at the top right quadrant	0.70	0.76	0.83
Cronbach's Alpha for all items	0.89	0.97	0.96

**Figure 3.**
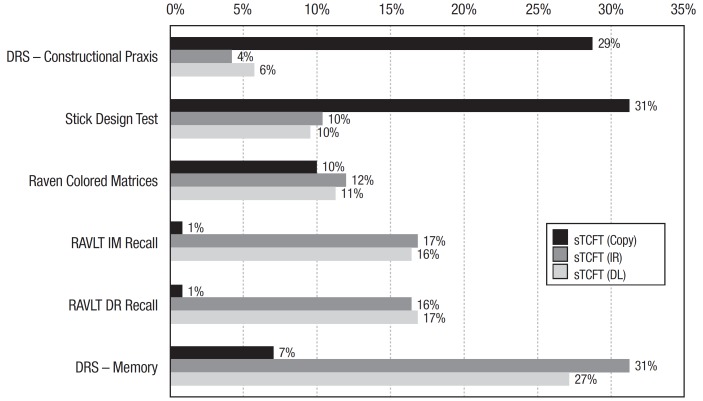
Shared variance (r²) between the "Simplified" Taylor Complex Figure Test components and measures of Intelligence, Visuospatial Abilities and Episodic

A significant pattern of correlations emerged between the sTCFT and other measures of Visuospatial Abilities and Episodic Memory. These associations are summarized in Figure 3. 

sTCFT: Simplified Taylor Complex Figure Test; IR: Immediate Recall; DR: Delayed Recall; DRS: Dementia Rating Scale; RAVLT: Rey Auditory- Verbal Learning Test.

## DISCUSSION

The present study introduces a preliminary analysis of the psychometric characteristics of the complex figure test, adapted for use in older adults with low formal education. Our analyses showed reliability (inter-rater agreement on the test scoring and high internal consistency), construct validity (convergent and divergent correlations) and criterion-related validity (significant differences between normal aging subjects and clinical groups). Taken together these results suggest that the sTCFT is an adequate test for the assessment of older adults with low formal education. 

 The analyses of group comparisons on the sTCFT among the three clinical groups and normal aging controls are coherent with the disproportionate episodic memory impairment presented by amnestic MCI and AD patients (large effect sizes were found on both immediate and delayed recall trials), while deficits on the copy procedure were significantly milder and circumscribed in MDaMCI and AD. Overall, these findings suggests that impairment on the sTCFT recall trials might be useful for characterizing aMCI, MDaMCI and AD. Future studies should be conducted to develop normative data and cut-off scores for the test. 

The sTCFT showed a high internal consistency for the Copy (0.89), Immediate Recall (0.97) and Delayed Recall (0.96) components, providing evidence of test reliability. Studies usually show higher internal consistency values for recall than copy trials [21]. The inter-rater reliability showed higher agreement for recall trials (correlations between examiners' scores of 0.96 and 0.97) compared to copy (correlation of 0.89) trials. This might have occurred due to the randomized selection of participants for this procedure: those with very low educational background and or illiteracy as well as AD were included, and usually produce a more disorganized copy, thereby increasing the variability of specific item quotation. When compared to previous studies in healthy subjects with higher formal education, our results indicate lower agreement between judges. For instance, a recent study^22^ reported a correlation coefficient of 0.99 when scoring a modified version of the Taylor Complex Figure Test in a younger and more educated sample. Nonetheless, the finding remain within recommended parameters of reliability. 

The Copy trial was associated with the Stick Design Test and DRS Constructional Praxis, which provides evidence of construct validity as a measure of spatial information processing. Other studies adopting different measures of drawing tasks have found significant associations between the copy trials and other measures of spatial processing, such as constructional praxis and object assembly^23^ and judgment of angle orientation.^24^ These results indicated that drawing is one of the many paradigms that neuropsychologists could adopt to assess Visuospatial Abilities. Drawing tasks also have important advantages in clinical practice, as discussed in a review,^24^ since they involve complex and multidimensional processes that can yield different kinds of quantitative and qualitative information regarding constructional praxis, visuospatial attention and visual memory. We also found significant associations between the memory components of the sTCFT and other memory measures, such as the DRS memory subscale and RAVLT immediate and delayed recall tests. Previous studies have documented significant correlations between the complex figure recall and the RAVLT.^26^ Combining the data between copy and recall conditions, we found validity for the two hypothesized constructs (visuospatial abilities and episodic memory). 

The only neuropsychological measure selected for the construct validity analysis that showed a similar association between the copy and recall trials was the Raven Progressive Matrices Test, our measure of Intelligence. We expected a stronger association between the test score and sTCFT copy than recall, given the spatial demands of the test. However, the pattern was the same across all three test conditions. This unexpected result may reflect sample characteristics. A study in a Latino population^27^ showed a significant association between the Rey Complex Figure Test copy and recall with the Raven Standard Progressive Matrices total score. Specifically, characteristics of this population, especially the cultural-educational background, might strengthen the relationship between fluid intelligence and episodic memory recall, perhaps due to compensational strategies for a markedly difficult task in individuals with lower formal education. Future studies should address this particular issue. 

Our preliminary results on the sTCFT should be viewed in light of the study limitations. The sample was heterogeneous and contained a large proportion of clinical subjects on the MCI-AD spectrum yet relatively few normal aging elderly. In this sense, a mixed sample with disproportionate groups was involved. This precluded calculation of the parameters of interpretation or the contribution of different sociodemographic aspects to the test performance. These procedures in healthy participants or mixed clinical groups might be biased by specific patterns of variance characteristics of these populations. Future studies should be conducted to replicate our findings in other clinical groups and investigate the test characteristics in a larger sample of healthy older adults. In addition, other aspects of validity and reliability (such as test-retest reliability) beyond the scope of this study should be addressed in future research. 
